# Association between Insomnia and Metabolic Syndrome in a Chinese Han Population: A Cross-sectional Study

**DOI:** 10.1038/s41598-017-11431-6

**Published:** 2017-09-07

**Authors:** Yan Wang, Tian Jiang, Xiaoqiang Wang, Jianrong Zhao, Jinwang Kang, Min Chen, Haifang Wang, Lili Niu, Youxin Wang, Yong Zhou, Jing Wu, Hui Fu, Zhaoyang Cai, Zemin Li, Junzheng Chen

**Affiliations:** 1 Intensive Care Unit, the Second Affiliated Hospital of Hebei North Medical College, Xuanhua, Hebei, 075100 China; 2 Research Center for Translational Medicine, the Affiliated Wenling Hospital of Wenzhou Medical University, Wenling, Zhejiang, 317500 P.R. China; 3 Department of Internal Medicine, the Second Affiliated Hospital of Hebei North Medical College, Xuanhua, Hebei 075100 China; 4 Beijing Key Laboratory of Clinical Epidemiology, School of Public Health, Capital Medical University, Beijing, 100069 China; 5 Beijing Institute of Heart, Lung and Blood Vessel Diseases, Beijing Anzhen Hospital, Capital Medical University, Beijing, 100029 China; 6 Beijing Rec Data Technology Co., Lmt. Beijing, Beijing, 102600 China

## Abstract

The association between insomnia and metabolic syndrome remains unclear, especially among different-aged groups. A cross-sectional study with 8017 participants was performed to identify whether insomnia was associated with metabolic syndrome or not. Demographic characteristics, lifestyles and other risk factors were collected using a predesigned, semi-structured, self-administered questionnaire, and physical examinations were conducted by certificated community physicians. Insomnia was not independently associated with metabolic syndrome across all subjects; however, the association between insomnia and metabolic syndrome was statistically significant in the male group (odds ratio (OR): 1.35, 95% confidence interval (CI): 1.02–1.77) and the middle-aged group (OR: 1.40, 95% CI: 1.09–1.79) but not in the female group, the young adult group or the older group. Analyses of the individual components of metabolic syndrome revealed that insomnia was independently associated with raised blood pressure (OR: 1.24, 95% CI: 1.05–1.43) and low high-density lipoprotein cholesterol (HDL-c) (OR: 1.16, 95% CI: 1.01–1.33). Insomnia was also independently associated with the severity of metabolic abnormalities (OR: 1.17, 95% CI: 1.03–1.32). This study demonstrates an independent association between insomnia and metabolic syndrome in males and middle-aged participants, which suggests that treatment for insomnia will contribute to the prevention of metabolic syndrome in males and the middle-aged population.

## Introduction

Metabolic syndrome is a complex of interrelated risk factors for cardiovascular disease and diabetes. The factors include obesity (particularly central adiposity), raised blood pressure, dysglycemia, low high-density lipoprotein cholesterol (HDL-c) levels and elevated triglyceride levels^1^. Metabolic syndrome is one of the major pandemics that affect health across the globe^2^. The prevalence of metabolic syndrome is approximately 30% in the world^3–5^, and ranges from 7% to 32% in China^6–10^.

Insomnia is a subjective feeling of difficulty initiating sleep, difficulty maintaining sleep, waking up too early, non-restorative sleep or poor quality of sleep^11^. With the development of society and the economy, more and more people suffer from insomnia due to the increasing pressures of life and work^12, 13^. Insomnia is a serious public health issue. The worldwide prevalence of insomnia in the general population ranges from 8% to 40%^14^, and the annual prevalence of insomnia symptoms in the American adult population ranges from 35% to 50%^15^.

Some studies have shown that insomnia is associated with metabolic syndrome^16–18^. A 1-year follow-up study reported that insomnia was a significant predictor of metabolic syndrome. The risk of metabolic syndrome incidence was 2.17 (95% CI: 1.13–4.15) for those with insomnia compared to those without insomnia^16^. However, several studies have shown that insomnia is not related to metabolic syndrome^19–21^. Therefore, it is not yet conclusive whether insomnia is associated with metabolic syndrome or not. Consequently, we hypothesized that insomnia may be associated with metabolic syndrome in Chinese populations. The present study aimed to evaluate the association between insomnia and metabolic syndrome in a Chinese adult population, and, furthermore, to explore the association among participants stratified by age and sex, considering that both metabolic syndrome and insomnia are associated with age and gender^8, 11, 17^.

## Results

### Characteristics of participants according to the status of metabolic syndrome

**Figure 1:**
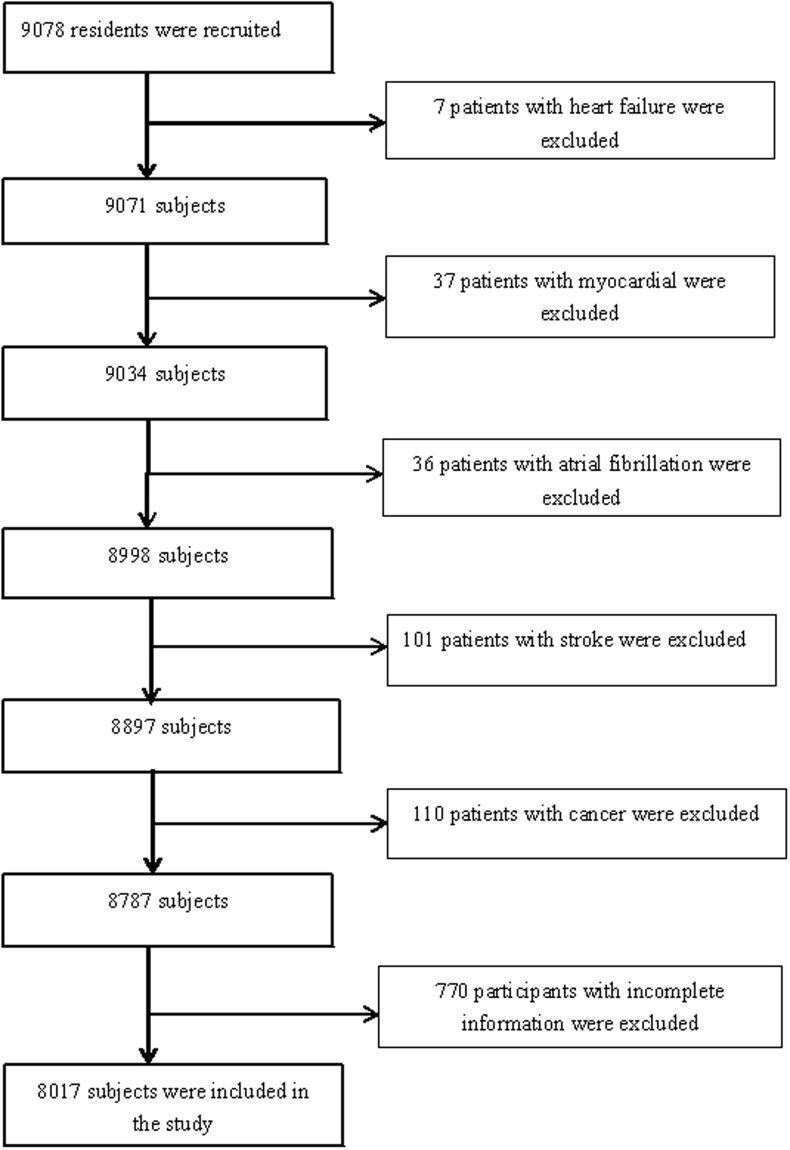
Flow Chart of the Enrolled Participants Meet the Requirements.

Of the 9078 participants, 8017 individuals were included in the final statistical analysis, while 1061 individuals were excluded, including 7 patients with heart failure, 37 patients with myocardial infarction, 36 patients with atrial fibrillation, 101 patients with stroke, 110 patients with cancer and 770 participants with incomplete information (Fig. 1). The study consisted of 4152 men (51.79%) and 3865 women (48.21%), with a mean age of 42.08 ± 12.95 years. There were 3927, 3150 and 940 participants aged less than 40 years, 40–59 years and 60 years or older, respectively. The prevalence of metabolic syndrome was 29.70% among all participants, 34.61% in males and 24.24% in females. The prevalence of metabolic syndrome was 19.79%, 35.37% and 52.13% in the young adult group (less than 40 years old), the middle-aged group (40–59 years old) and the older group (60 years old or older), respectively. The differences in gender, age, education degree, income, smoking status, drinking status, salt intake, physical activity and body mass index (BMI) were statistically significant (*P* < 0.05) between the participants with metabolic syndrome and those without metabolic syndrome. Insomnia (AIS ≥ 6) was found in 1019 subjects (12.71%), of which 366 (4.57%) were diagnosed with metabolic syndrome and 653 (8.15%) were not diagnosed with metabolic syndrome (*P* *<* 0.05) (Table 1).

**Table 1:** Characteristics of participants according to the status of metabolic syndrome.

Variables	Total (N = 8017)	Metabolic syndrome		*P*
		Present (n = 2381)	Absent (n = 5636)	
**Gender·n(%)**				<0.01
male	4152	1437(34.61)	2715(65.39)	
female	3865	944(24.42)	2921(75.58)	
**Age·n(%)**				<0.01
<40 years	3927	777(19.79)	3150(80.21)	
40–59 years	3150	1114(35.37)	2036(64.63)	
≥60 years	940	490(52.13)	450(47.87)	
**Education degree·n(%)**				<0.01
illiteracy or primary school	291	156(53.61)	135(46.39)	
middle school	2788	1111(39.85)	1677(60.15)	
high school or higher	4938	1114(22.56)	3824(77.44)	
**Income·n(%)**				<0.01
≤¥3000	3032	1050(34.63)	1982(65.37)	
¥3001–5000	4371	1162(26.58)	3209(73.42)	
>¥5000	614	169(27.52)	445(72.48)	
**Smoking status·n(%)**				<0.01
never	5681	1445(25.44)	4236(74.56)	
current	2061	800(37.82)	1261(61.18)	
former	275	136(49.45)	139(50.55)	
**Drinking status·n(%)**				<0.01
none	5347	1456(27.23)	3891(72.77)	
<1 standard quantity/day	1204	390(32.39)	814(67.61)	
≥1 standard quantity/day	1466	535(36.49)	931(63.51)	
**Salt intake·n(%)**				<0.01
low	1719	457(26.59)	1262(73.41)	
medium	4106	1175(28.62)	2931(71.38)	
high	2192	749(34.17)	1443(65.83)	
**Physical activity·n(%)**				<0.01
very active	4154	1323(31.85)	2831(68.15)	
moderately active	767	185(24.12)	582(75.88)	
inactive	3096	873(28.20)	2223(71.80)	
**BMI(kg/m** ^**2**^ **)**	24.53 ± 3.74	23.16 ± 23.08	27.78 ± 27.65	<0.01
**AIS·n(%)**				<0.01
non-insomnia(<6 points)	6998	2015(28.79)	4983(71.21)	
insomnia(≥6 points)	1019	366(35.92)	653(64.08)	

### Association between insomnia and metabolic syndrome in adults from the Jidong community

Insomnia was associated with metabolic syndrome in the crude model (OR: 1.39, 95% CI: 1.21–1.59) and the model adjusted for age and gender (OR: 1.18, 95% CI: 1.02–1.37). After adjusting for age, gender, education degree, income, smoking status, drinking status, salt intake, physical activity and BMI, the association between insomnia and metabolic syndrome was not statistically significant among all subjects (OR: 1.18, 95% CI: 0.98–1.41). In the stratified analysis, insomnia was associated with metabolic syndrome after adjusting for all potential confounding factors in the male group (OR: 1.35, 95% CI: 1.02–1.77) and in the middle-aged group (OR: 1.40, 95% CI: 1.09–1.79), but this association was not observed in the female group (OR: 1.01, 95% CI: 0.78–1.29), in the young adult group (OR: 0.88, 95% CI: 0.60–1.30) or in the older adult group (OR: 1.08, 95% CI: 0.73–1.61) (Table 2).

**Table 2:** Association between insomnia and metabolic syndrome in adults from the Jidong community.

	OR	95% CI	*P*
**Model 1**	1.39	(1.21, 1.59)	<0.01
**Model 2**	1.18	(1.02, 1.37)	0.03
**Model 3**	1.18	(0.98, 1.41)	0.08
**Model 4**			
Male	1.35	(1.02, 1.77)	0.03
Female	1.01	(0.78, 1.29)	0.96
**Model 5**			
<40 years	0.88	(0.60, 1.30)	0.52
40–59 years	1.40	(1.09, 1.79)	0.01
≥60 years	1.08	(0.73, 1.61)	0.71

Model 1. crude model.

Model 2. adjusted for age and gender.

Model 3. adjusted for age, gender, education degree, income, smoking, drinking, salt intake, physical activity and BMI.

Model 4. adjusted for age, education degree, income, smoking, drinking, salt intake, physical activity and BMI.

Model 5. adjusted for gender, education degree, income, smoking, drinking, salt intake, physical activity and BMI.

### Association between insomnia and the components of metabolic syndrome in adults from the Jidong community

Analyses of the individual components of metabolic syndrome revealed that insomnia was independently associated with raised blood pressure (OR: 1.22, 95% CI: 1.05–1.43) and low HDL-c levels (OR: 1.16, 95% CI: 1.01–1.33) but not with central adiposity (OR: 1.13, 95% CI: 0.93–1.38), dysglycemia (OR: 1.02, 95% CI: 0.86–1.21) or elevated triglyceride levels (OR: 1.10, 95% CI: 0.94–1.28) in all participants after adjusting for all potential confounding factors. In the stratified analysis, insomnia was associated with raised blood pressure (OR: 1.52, 95% CI: 1.22–1.88) and central adiposity (OR: 1.46, 95% CI: 1.11–1.91) in the middle-aged group after adjusting for all potential confounding factors (Table 3).

**Table 3:** Association between insomnia and the components of metabolic syndrome in adults from the Jidong community.

	Central adiposity			Raised Blood pressure			Dysglycemia			Low HDL-c			Elevated Triglycerides		
	OR	95% CI	*P*	OR	95% CI	*P*	OR	95% CI	*P*	OR	95% CI	*P*	OR	95% CI	*P*
**Model 1**	1.38	(1.21, 1.57)	<0.01	1.34	(1.18, 1.53)	<0.01	1.32	(1.13, 1.55)	<0.01	1.24	(1.09, 1.42)	<0.01	1.11	(0.96, 1.27)	0.15
**Model 2**	1.11	(0.97, 1.28)	0.13	1.20	(1.03, 1.38)	0.02	1.03	(0.87, 1.22)	0.73	1.17	(1.02, 1.33)	0.03	1.12	(0.97, 1.30)	0.13
**Model 3**	1.13	(0.93, 1.38)	0.22	1.22	(1.05, 1.43)	0.01	1.02	(0.86, 1.21)	0.83	1.16	(1.01, 1.33)	0.04	1.10	(0.94, 1.28)	0.25
**Model 4**															
Male	1.24	(0.91, 1.69)	0.17	1.25	(0.99, 1.59)	0.06	1.05	(0.82, 1.35)	0.70	1.19	(0.95, 1.48)	0.13	1.12	(0.90, 1.41)	0.32
Female	1.05	(0.80, 1.36)	0.74	1.15	(0.93, 1.42)	0.20	0.98	(0.77, 1.26)	0.90	1.15	(0.96, 1.38)	0.14	1.00	(0.81, 1.25)	0.98
**Model 5**															
<40years	0.90	(0.62, 1.31)	0.59	0.91	(0.67, 1.22)	0.52	0.95	(0.62, 1.46)	0.81	1.13	(0.89, 1.44)	0.32	0.77	(0.56, 1.06)	0.10
40–59years	1.46	(1.11, 1.91)	0.01	1.52	(1.22, 1.88)	<0.01	1.09	(0.87, 1.37)	0.47	1.13	(0.92, 1.38)	0.24	1.18	(0.95, 1.46)	0.33
≥60years	0.90	(0.54, 1.50)	0.70	1.11	(0.75, 1.65)	0.60	0.98	(0.69, 1.39)	0.91	1.33	(0.94, 1.89)	0.11	1.21	(0.86, 1.71)	0.28

Model 1. crude model.

Model 2. adjusted for age and gender.

Model 3. adjusted for age, gender, education degree, income, smoking, drinking, salt intake, physical activity and BMI.

Model 4. adjusted for age, education degree, income, smoking, drinking, salt intake, physical activity and BMI.

Model 5. adjusted for gender, education degree, income, smoking, drinking, salt intake, physical activity and BMI.

### Association between insomnia and the severity of metabolic abnormalities in adults from the Jidong community

Insomnia was associated with the severity of metabolic abnormalities (OR: 1.17, 95% CI: 1.03–1.32) in all participants after adjusting for age, gender, education degree, income, smoking status, drinking status, salt intake, physical activity and BMI. In the stratified analysis, this association was also found in the male group (OR: 1.27, 95% CI: 1.03–1.54) and in the middle-aged group (OR: 1.37, 95% CI: 1.15–1.63), while the association was not observed in the female group (OR: 1.10, 95% CI: 0.93–1.28), the young adult (OR: 0.93, 95% CI: 0.76–1.16) or the older adult group (OR: 1.20, 95% CI: 0.89–1.61) after adjusting for potential confounding factors (Table 4).

**Table 4:** Association between insomnia and the severity of metabolic abnormalities in adults from the Jidong community.

	OR	95% CI	*P*
**Model 1**	1.39	(1.24, 1.56)	<0.01
**Model 2**	1.16	(1.03, 1.31)	0.01
**Model 3**	1.17	(1.03, 1.32)	0.01
**Model 4**			
Male	1.27	(1.03, 1.54)	0.02
Female	1.10	(0.93, 1.28)	0.28
**Model 5**			
<40 years	0.93	(0.76, 1.16)	0.53
40–59 years	1.37	(1.15, 1.63)	<0.01*
≥60 years	1.20	(0.89, 1.61)	0.23

Model 1. crude model.

Model 2. adjusted for age and gender.

Model 3. adjusted for age, gender, education degree, income, smoking, drinking, salt intake, physical activity and BMI.

Model 4. adjusted for age, education degree, income, smoking, drinking, salt intake, physical activity and BMI.

Model 5. adjusted for gender, education degree, income, smoking, drinking, salt intake, physical activity and BMI.

## Discussion

Based on the large population-based sample, this study showed that there was an independent association between insomnia and metabolic syndrome in males and in middle-aged people; insomnia was also associated with raised blood pressure and low HDL-c in all participants. For middle-aged people, insomnia was associated with raised blood pressure and central adiposity, which are major risk factors for cardiovascular disease and diabetes. To our knowledge, the current study is the first to investigate the relationship between insomnia and metabolic syndrome in the mainland of China. The study further validated the association between insomnia and metabolic syndrome, as this association has been reported to be inconsistent in different populations^16, 18, 20, 21^.

In the current study, we found that the association between insomnia and metabolic syndrome was not statistically significant among all subjects after adjusting for potential confounding factors. This result was similar to that of a previous study, in which Gu *et al*. examined the relationship between lifestyles and metabolic syndrome, and found that insomnia was not independently correlated to metabolic syndrome^21^. However, another study that included 3936 adults showed that metabolic syndrome was closely associated with insomnia (OR: 1.23, 95% CI: 1.03–1.48)^18^. The absence of significant association in the result from model 3 in the current study might be due to collinearity between independent variables or the interaction of intermediate and confounding factors, as well as the loss of statistical power due to introduction of more confounding factors. Further well-designed epidemiological investigations are required to clarify this association.

The current study explored the association between insomnia and metabolic syndrome in people of different ages. We found that insomnia was independently associated with metabolic syndrome in middle-aged people. This association might be because middle-aged people bear double pressures of society and family and because this age group often lacks physical activities and has unhealthy eating habits, all of which impact both insomnia and metabolic syndrome^22, 23^. Additionally, an increase in sleep problems was already evident in midlife, as there was an increased incidence of other diseases as well as mood symptoms, both of which might exert an effect on sleep quality either directly or via side-effects of the associated medications. Weight changes during midlife might also affect sleep quality^24^, and obesity is a component of metabolic syndrome. In our study, an independent association between insomnia and metabolic syndrome was not observed in the young adults or older adults after adjusting for potential confounding factors. However, Chen *et al*. reported that participants with insomnia had more than twice the incidence of metabolic syndrome than did individuals without insomnia in an older population^16^. This difference might be caused by the diagnostic criteria of insomnia and metabolic syndrome or by the absence of statistical power when controlling for multiple confounding factors.

The present study showed that the independent association between insomnia and metabolic syndrome was observed in the male group. Previous studies that examined the association between insomnia and metabolic syndrome focused mainly on the general population and females^17, 19, 20^. This is the first study to indicate gender differences in the association between insomnia and metabolic syndrome. Our results differ from those of Troxel *et al*., who reported that specific symptoms of insomnia (difficulty falling asleep and “unrefreshing” sleep) but not insomnia syndrome were significant predictors of the development of metabolic syndrome, and these effects were similar among men and women^19^. The inconsistence remains unexplained, but it could be explained in part by the fact that both metabolic syndrome and insomnia are associated with gender^8, 11, 17^. This inconsistency might be explained by different estrogen levels between males and females and between those of postmenopausal and premenopausal women. Postmenopausal women have more sleep disturbances than premenopausal women^25, 26^. Additional studies on the association between insomnia and metabolic syndrome are required and should account for estrogen levels.

The mechanism of the association between insomnia and metabolic syndrome remains unclear. It has been reported that hypothalamic-pituitary-adrenal (HPA) axis hyperactivity plays a role in the pathogenesis of insomnia^11^, and activation of the HPA axis can lead to metabolic syndrome^27^. In addition, some studies have shown that sleep fragmentation or restriction leads to insulin resistance^28–31^, which plays a key role in the pathophysiology of metabolic syndrome^32^. Thus, chronic sleep debt may have modulatory effects on glucose metabolism and promote the development of metabolic syndrome.

Insomnia was found to be associated with raised blood pressure, which is similar to the results of the majority of published studies^33–38^. Benbir *et al*. reported that insomnia was significantly associated with the presence of hypertension after the adjustment for relevant risk factors^36^. Lewis *et al*. reported that individuals in a chronic insomnia cohort were at twice the risk for hypertension^33^. In addition, a meta-analysis supported that sleep continuity disturbance, early-morning awakening and combined symptoms of insomnia were associated with an increased risk of hypertension^34^. Several potential mechanisms have been suggested to explain the association between insomnia and raised blood pressure. Poor sleep quality is related to BMI^39^, while higher BMI increases the risk of hypertension^40^. The pathophysiology of insomnia is not yet fully understood. However, Roth *et al*. reported that increased activity of the HPA axis is associated with the pathology of chronic insomnia^11^. Furthermore, the 24-h mean adreno-cortico-tropic-hormone (ACTH) and cortisol secretions are significantly higher in insomniacs^41^, and elevated cortisol is known to raise blood presure^42, 43^.

An independent association between insomnia and low HDL-c was observed, which is consistent with previous results that showed that low HDL-c was an independent predictor of poor sleep^35^. However, Vozoris reported that insomnia symptoms were not associated with high triglycerides or low HDL-c^44^. Zhan *et al*. found no significant association between insomnia symptoms and high LDL-c, high triglycerides, or low HDL-c^45^. This difference might be caused by the sample size, age and region of the population. We observed that insomnia was independently associated with low HDL-c, which suggests that treatment for insomnia might improve the levels of HDL-c, which is a protective factor for cardiovascular disease.

Insomnia was independently associated with the severity of metabolic abnormalities. The risk of having one increased level of metabolic abnormalities in patients with insomnia was 17% higher than in those without insomnia, and the risk was relatively high in middle-aged insomniacs, up to 137%. Therefore, middle-aged patients with insomnia tend to suffer not only from metabolic syndrome but also from more severe metabolic abnormalities. The association is probably due to stressful work and unhealthy lifestyles of middle-aged people.

This study had several limitations. First, our study was a cross-sectional study; therefore, we were not able to evaluate the causality between insomnia and metabolic syndrome. Prospective studies with large sample sizes need to be conducted to explore the causal relationship between insomnia and metabolic syndrome. Second, the studied population was from a community in northern China, which restricts the generalization of the finding.

In conclusion, we observed an independent association between insomnia and metabolic syndrome in males and middle-aged subjects, and insomnia was also associated with some components of metabolic syndrome. Furthermore, insomniacs were prone to more severe metabolic abnormalities. These findings suggest that treatment for insomnia in males and middle-aged people might benefit the prevention of metabolic syndrome.

## Methods

### Ethics Statement

The study was conducted according to the guidelines of the Helsinki Declaration and was approved by the Ethics Committee of Jidong Oilfeld Inc. Medical Centers. Written informed consents were obtained from all participants.

### Study Subjects

The subjects were recruited from the Jidong community, Tangshan, which is a large modern city in the eastern part of Hebei Province^46, 47^. In brief, from July 2013 to August 2014, a total of 9078 residents aged 18–82 years who were not pregnant and were willing to participate in the study were recruited. Furthermore, we excluded subjects who met the following criteria: (i) patients with heart failure, myocardial infarction or atrial fibrillation; (ii) patients with stroke; (iii) patients with cancer; and (iv) participants with incomplete information. Ultimately, a total of 8017 participants were eligible and included in our study.

### Assessment of Metabolic Syndrome

Metabolic syndrome was diagnosed according to the 2005 IDF consensus worldwide definition of metabolic syndrome^1^. The severity of metabolic abnormalities was graded by the prevalence components of metabolic syndrome on a scale of 0 to 5.

### Assessment of Insomnia

Insomnia was assessed by the Athens Insomnia Scale (AIS)^48^, which is a psychometric self-assessment designed for quantifying sleep difficulty. It consists of 8 items: the first 5 refer to sleep induction, awakenings during the night, final awakening, total sleep duration and sleep quality, and the last 3 pertain to well-being, functioning capacity and sleepiness during the day. The each item is measured on a 0–3 numeric rating scale. Patients are asked to rate the severity of their insomnia, with 0 being “no problem” and 3 being “did not sleep at all”. The total score is from 0 to 24 points. Patients with scores of 6 and above are considered to have insomnia^49^.

### Assessment of Demographic Variables and the Risk Factors of Metabolic Syndrome

Subjects participated in face-to-face questionnaires and physical examinations. Information regarding each subject’s gender, age, education degree, income and lifestyles (smoking status, drinking status, salt intake and physical activity) was collected using a predesigned, semi-structured, self-administered questionnaire^46, 47^. Questionnaires were graded according to methods in previous studies^46, 50^. Education degree was categorized as “illiteracy or primary school”, “middle school” or “high school or higher”. Average income per person in a family was reported as “≤¥3000”, “¥3001–5000” or “>¥5000”. Smoking status was classified as “never”, “current” or “former”, and drinking status was classified as “none”, “<1 standard quantity/day” or “≥1standard quantity/day”. Salt intake was classified as “low (salt: <6 g/day)”, “medium (salt: 6–10 g/day)” or “high (salt: >10 g/day)”^50^. Physical activity was classified as “very active (exercise ≥150 min/week of moderate intensity or ≥75 min/week of vigorous intensity)”, “moderately active (exercise: 1–149 min/week of moderate intensity or 1–74 min/week of vigorous intensity)” or “inactive (exercise: none)”^46^. Physical examinations were conducted by certificated community physicians. An electronic balance was used to measure weight, and a meter rule was used to measure the height of the subjects; BMI was calculated as weight (kg) divided by the square of the height (m^2^). The measurements of waist circumference and blood pressure were reported previously^19, 51^.

### Biochemical Indicators

Blood samples were collected from the antecubital vein from subjects who fasted overnight and were centrifuged at a speed of 3000 r/min at room temperature; within four hours, the serum was tested using an automatic biochemical analyzer (Olympus AU400, Japan) in the central laboratory of the Jidong Oilfield Hospital. Biochemical indicators included fasting blood glucose, triglyceride and HDL-c levels.

### Data Management and Statistical Analyses

The data were handled and managed using the Ruichi Precision Medical Record System (RPMRS), which was developed to standardize, integrate, manage, and analyze precision medical data. The statistical analyses were performed using SAS software (Version 9.4, SAS Institute, Cary, North Carolina, USA). Continuous variables were described as the mean with standard deviation, and the intergroup differences were assessed using Student’s t-test or ANOVA. Categorical variables are described with percentages, and the intergroup differences were assessed using a χ^2^ test. A nonparametric test was used for comparisons of the ordinal variables. Logistic regression analyses were performed to adjust for potential confounding factors. The association between insomnia and metabolic syndrome was represented using the odds ratio (OR) and 95% confidence interval (CI). All statistical tests were two-sided, and *P* < 0.05 was considered significant.
